# The prevalence and risk factors of musculoskeletal disorders among Indonesian dental professionals

**DOI:** 10.3389/fresc.2025.1513442

**Published:** 2025-02-12

**Authors:** Erica Kholinne, Xarisa Azalia, Erika Putri Rahayu, Ira Juliet Anestessia, Nadifa Agil, Muchtar

**Affiliations:** ^1^ Faculty of Medicine, Universitas Trisakti, Jakarta, Indonesia; ^2^ School of Medicine and Health Sciences, Atma Jaya Catholic University of Indonesia, Jakarta, Indonesia

**Keywords:** musculoskeletal disorders, dentistry, occupational medicine, prevention, work-related musculoskeletal disorders

## Abstract

Dental professionals (DPs) are at significant risk of developing work-related musculoskeletal disorders (WMSDs). This study aimed to determine the prevalence of WMSDs among DPs and identify associated factors based on professional level. A cross-sectional analysis was conducted on 151 dental professionals, including dentists, dental nurses, and dental assistants, from various universities and health institutes in Indonesia. Data were collected using the Nordic Musculoskeletal Questionnaire. Associations between WMSD symptoms and qualitative data (demographics, job characteristics, and other factors) were examined using the Chi-square test, while quantitative data were analyzed using the independent *t*-test. The results showed that 96% of respondents (145 out of 151) had experienced WMSDs, with the back (68.2%), waist (66.9%), upper neck (60.9%), and lower neck (59.6%) being the most commonly affected regions. Muscle fatigue (53.6%) and pain (49.7%) were the most frequently reported symptoms. Triggering factors included unergonomic body posture (84.1%) and prolonged sitting (53.6%), while protective factors included rest (71.5%) and improved body posture (53%). Physical exercise was significantly associated with WMSDs (*p* < 0.001). This study highlights the high prevalence of WMSDs among dental professionals and underscores the need for ergonomic training, physical exercise, and equipment modification to mitigate these conditions. Limitations of the study include unequal distribution among professional groups and a gender imbalance, which may affect the generalisability of the findings.

## Introduction

Work-related musculoskeletal disorders (WMSDs) are a common type of work-related illness, accounting for approximately 48% of all cases (1). WMSDs can cause work-related disabilities, reducing productivity and increasing costs (1, 2). WMSDs are a type of multifactorial alteration of muscles and tendons due to repetitive movements and physical tension in the limbs while working caused by the position or movement involved (2). Work-related musculoskeletal disorders (WMSDs) refer to injuries affecting the human support system, such as bones, cartilage, muscles, ligaments, tendons, blood vessels, or nerves. These injuries are primarily caused or worsened by work tasks and the working environment (3–6). Musculoskeletal disorders are classified as work-related when both the working environment and the performance of work significantly contribute to the condition, exacerbate it, or prolong its duration (7).

Health workers, particularly dentists, nurses, doctors, and surgeons, are more susceptible to developing WMSDs. In dentistry, many WMSDs are linked to cumulative trauma, often manifesting as repetitive strain injuries. Studies have shown that dentists are prone to work-related musculoskeletal complaints, with pain being the most common complaint, affecting the neck the most (8–16). Common examples of work conditions that can lead to WMSDs include lifting heavy objects regularly, being exposed to whole-body vibration daily, performing overhead work routinely, working with the neck in a constantly flexed position, or engaging in repetitive forceful tasks (7). Dental practitioners are at risk for repetitive strain injuries due to activities such as gripping and using slender instruments repetitively (e.g., for plaque removal and cavity preparation) and using vibratory instruments like handpieces and ultrasonic scalers (17, 18). The nature of dental work also contributes to WMSD risks, as practitioners frequently sustain awkward postures and non-neutral wrist positions.

A cross-sectional study of 536 dentists in India conducted by Kumar et al. (12) found that all respondents had work-related musculoskeletal complaints, with pain being the most common complaint (99.06%). The neck was the most frequently affected body part. Similarly, Phedy et al. (14) found that 63.5% of 241 Indonesian dentists reported musculoskeletal complaints. Prolonged static postures, repetitive movements, and awkward body positions during patient care are the primary risk factors for developing musculoskeletal disorders (11, 16).

Musculoskeletal disorders can adversely affect patient care quality and work productivity and increase work absence. Therefore, it is crucial to understand musculoskeletal disorders in DPs and develop effective prevention strategies to improve their health and work productivity (11, 12, 19, 20). This research aimed to investigate the prevalence of WMSDs in DPs, comprising dentists, dental nurses, and dental assistants, and to identify the associated factors for WMSD based on professional level.

## Methods

This research was a cross-sectional study conducted from December 2023 to March 2024. The study collected data on potential risk factors (e.g., demographic information, BMI, exercise frequency) as independent variables, also professional levels and job characteristics (type of profession, duration of working hours and overtime, work position) as possible mediators or moderators. To determine the appropriate sample size for the study, we used a power of 80%, a two-sided significance level of 0.05, and an anticipated difference in incidence rates of 10% (63.5% and 53.5%). The incidence rate of 63.5% was derived from a previous study by Phedy et al. (14). Based on these parameters, the study required a minimum of 25 patients in each group to meet the sample size requirement. The inclusion criteria are dentists, dental nurses, and dental assistants. The exclusion criteria are dentists, dental nurses, and dental assistants with a history of musculoskeletal surgery. The study was without a control arm. The Indonesian version of the Nordic Musculoskeletal Questionnaire was utilized (21, 22). The questionnaire consisted of 27 Likert scale items, to identify areas of the body that have caused musculoskeletal problems in the past 12 months. The musculoskeletal disorders evaluated in this study were pain, stiffness, fatigue, discomfort, “clicking” sounds from joints, and neurological symptoms such as tingling and numbness. We employed an independent *t*-test to examine the association between WMSD and factors such as gender, age, weekly working hours, overtime hours, and working position. To assess the correlation between musculoskeletal disorders and BMI, exercise frequency, educational level, and type of profession, we conducted a one-way ANOVA test. Statistical analysis was conducted using the SPSS version 29 software, with a *p*-value of <0.05 considered statistically significant.

### Statistical analysis

The primary objective of this study was to develop a descriptive and inferential statistical framework to assess the relationship between risk factors (e.g., gender, age, weekly working hours, overtime hours, BMI, exercise frequency, educational level, and type of profession) and the prevalence of work-related musculoskeletal disorders (WMSDs) as binary outcomes. To address this objective, we conducted a cross-sectional study design without a control arm to explore associations and develop predictive insights.

In the unadjusted analyses, independent *t*-tests were used to examine the association between WMSDs and continuous variables such as gender, age, weekly working hours, overtime hours, and working position. Additionally, one-way ANOVA tests were conducted to evaluate the correlation between WMSDs and categorical variables such as BMI, exercise frequency, educational level, and type of profession.

For multivariable analysis, a backward elimination variable selection method was utilized to identify the most significant predictors of WMSDs. Interaction effects between variables, such as type of profession and working hours, were explored to determine potential effect modifications.

All statistical analyses were conducted using SPSS version 29 software, with a *p*-value of <0.05 considered statistically significant. Results were summarized as mean ± standard deviation (SD) or percentages for descriptive statistics, and *p*-values for inferential statistics. Additional sensitivity analyses were conducted to validate the findings from the primary analyses using alternative stratifications of professional level and exercise frequency.

## Results

**Figure 1:**
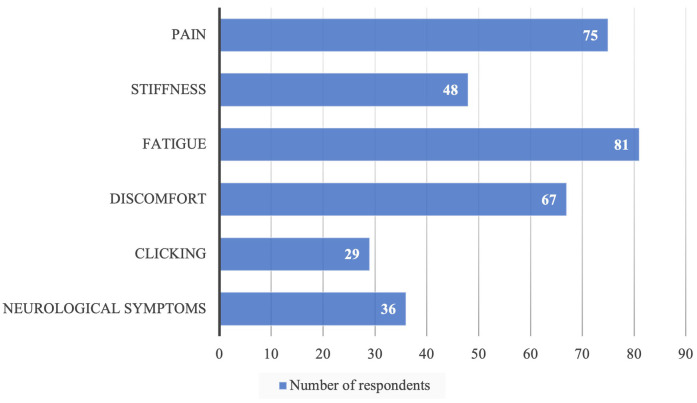
Symptoms of musculoskeletal disorders.

This study included 151 dental health practitioners. Table 1 provides an overview of the respondents' characteristics. On average, respondents worked 29.7 h per week, with 56.9% having overtime hours, averaging 7.3 h per week. 73.5% of respondents mostly worked in a sitting position, and the remaining respondents worked in a standing position. Among the 151 respondents, 26.5% did not have an exercise habit, 28.5% exercised less than once a week, 31.8% exercised 1–2 times a week, 9.9% exercised 3–4 times a week, and the remaining respondents exercised more than four times a week. Of the 151 respondents, 145 experienced musculoskeletal disorders as described in Table 2. The Nordic musculoskeletal questionnaire indicates that the back (68.2%) and waist (66.9%) had the highest proportion of musculoskeletal disorders, followed by the upper neck (60.9%) and lower neck (59.6%). Fatigue (53.6%) and pain (49.7%) were the most common symptoms of musculoskeletal disorders (Figure 1). The study also found that unergonomic body posture when working (84.1%) and prolonged sitting (53.6%) were the most aggravating factors (Table 3). On the other hand, taking a short break (71.5%) and improving body posture (53%) were the most influential factors in alleviating musculoskeletal disorders (Table 4). Based on a professional level, dentists had the highest burden of experiencing WMSD, followed by dental nurses and dental assistants (Table 5). According to Table 6, only physical exercise significantly influenced WMSD (sig < 0.001) among dental health practitioners.

**Table 1:** Socio-Demographic characteristics of study respondents.

Variables	Value
Gender (*n*, %)	
Male	21, 13.9
Female	130, 86.1
Age (*n*, %)	
≤30	84, 55.6
>30	67, 44.3
Body mass index (*n*, %)	
≤23	65, 43
>23	86, 56.9
Educational level (*n*, %)	
Bachelor	117, 77.5
Master	31, 20.5
Doctorate	3, 2
Type of profession	
Dental assistant	36, 23.8
Dental nurse	42, 27.8
Dentist	73, 48.3
Working hours per week, mean ± SD (hours)	29.7 ± 18.1
Respondents with overtime hours (*n*, %)	86, 56.9
Respondents without overtime hours (*n*, %)	65, 43
Overtime hours per week, mean ± SD (hours)	7.3 ± 5.7
Working position (*n*, %)	
Sitting	111, 73.5
Standing	40, 26.5
Exercise frequency (*n*, %)	
<once a week	43, 28.5
1–2 times	48, 31.8
3–4 times	15, 9.9
>4 times	5, 3.3
No exercise	40, 26.5

*n*, number of respondents, %, percentage; SD, standard deviation.

**Table 2:** Prevalence of musculoskeletal disorders (pain, stiffness, fatigue, discomfort, clicking, and neurological complaints) according to the nordic musculoskeletal questionnaire.

Body region	Pain level			Frequency (*n*) [number of respondents with complaints of moderate pain, pain, and very sick]	Proportion (%)
	Moderate pain	Pain	Very sick		
0 = Upper neck	75	14	3	92	60.9
1 = Lower neck	65	21	4	90	59.6
2 = Left shoulder	49	21	1	71	47.0
3 = Right shoulder	51	25	4	80	53.0
4 = Left upper arm	37	2	0	39	25.8
5 = Back	69	27	7	103	68.2
6 = Right upper arm	36	8	2	46	30.5
7 = Waist	58	34	9	101	66.9
8 = Buttock	46	13	2	61	40.4
9 = Bottom	33	15	1	49	32.5
10 = Left elbow	18	0	0	18	11.9
11 = Right elbow	20	2	0	22	14.6
12 = Left lower arm	30	0	0	30	19.9
13 = Right lower arm	31	2	1	34	22.5
14 = Left wrist	25	7	0	32	21.2
15 = Right wrist	48	16	1	65	43.0
16 = Left hand	31	1	0	32	21.2
17 = Right hand	46	4	1	51	33.8
18 = Left thigh	28	5	1	34	22.5
19 = Right thigh	25	3	2	30	19.9
20 = Left knee	33	5	3	41	27.2
21 = Right knee	34	4	2	40	26.5
22 = Left calf	32	11	4	47	31.1
23 = Right calf	33	10	3	46	30.5
24 = Left ankle	21	8	2	31	20.5
25 = Right ankle	19	8	1	28	18.5
26 = Left foot	30	5	1	36	23.8
27 = Right foot	24	6	0	30	19.9
Total respondents with musculoskeletal disorders (*n*, %):		145, 96%			

*n*, number of respondents; %, percentage.

**Table 3:** Factors that aggravate musculoskeletal disorders.

Aggravating factors	*n*, %
Prolonged sitting	81, 53.6
Incorrect posture	127, 84.1
Rotation	13, 8.6
Lifting	24, 15.9
Driving	7, 4.6
Trauma	9, 6

*n*, number of respondents; %, percentage.

**Table 4:** Factors that alleviate musculoskeletal disorders.

Alleviating factors	*n*, %
Correct posture	80, 53
Work pause	108, 71.5
Exercise	59, 39.1
Analgesic	33, 21.9
Sitting	28, 18.5
Bracing	2, 1.3
Rest	29, 19.2

*n*, number of respondents; %, percentage.

**Table 5:** Percentage of respondents with musculoskeletal disorders based on profession.

Profession	Respondents experiencing musculoskeletal disorders (*n*, %)
Dental assistant	34, 94.4
Dental nurse	40, 95.2
Dentist	71, 97.2

*n*, number of respondents; %, percentage.

**Table 6:** Association between musculoskeletal disorders and baseline participant characteristics.

Characteristics	Musculoskeletal disorders (n)	Percentage (%)	*P*-value
Gender			
Man	20	95.2	0, 843
Woman	125	96.1	
Age			
≤30	81	96.4	0,612
>30	64	95.5	
Body Mass Index (BMI)			
≤23	62	95.3	0.330
>23	83	96.5	
Level of education			
Bachelor	112	95.7	0.682
Master	30	96.7	
Doctorate	3	100	
Type of profession			
Co-assistant	34	94.4	0.455
Nurse	40	95.2	
Dentist	71	97.2	
Working hours per week			
≤40 h	106	95.4	0,562
>40 h	39	97.5	
Respondents with overtime hours	85	98.8	0.348
Working Position			
Sitting	108	97.2	0.185
Standing	37	92.5	
Exercise Frequency			
<1× per week	41	27.15	<0.001
1–2× per week	48	31.78	
3–4× per week	14	9.27	
>4× per week	3	1.98	
No Exercise	39	25.82	

*n*, number of respondents; %, percentage.

## Discussion

This study investigated the prevalence of work-related musculoskeletal disorders (WMSDs) among dentists, dental nurses, and dental assistants. Data were collected on potential risk factors, including demographic information, body mass index (BMI), and exercise frequency, which were analyzed as independent variables. Additionally, professional levels and job characteristics—such as type of profession, working hours, overtime, and work posture—were examined as potential mediators or moderators. The study revealed that 96% of respondents experienced WMSDs, which indicates that dental practitioners are vulnerable to WMSDs. This result also aligns with previous studies where DPs, which consisted of nurses, doctors, surgeons, and dental health workers, was considered vulnerable to WMSD (8–16).

This study found a higher prevalence rate of WMSDs among DPs than in previous studies. A study by Phedy et al. (14) found a WMSD prevalence rate of 63.5% among young dentists in Indonesia. The main difference between our study and theirs is that we included dentists, dental nurses, and dental assistants. Our study found a prevalence rate higher than that of several other countries, including 95% in Cameroon (23), 94% in a study of 120 dentists in Türkiye (24), 92% among 450 dentists and dental assistants in Germany (25), 87.2% in Australia (26), 86.5% in Lithuania (27), 85.6% in China (28), 81.4% in Brazil (29), 73.3% in India (30), 62% in Greece (31), 59.2% in Saudi Arabia (32), and 42% in the UK (33). The results of our study are consistent with a study in the Czech Republic, which found a WMSD prevalence rate of (34). These findings suggest that musculoskeletal disorders are relatively high and should not be underestimated by DPs.

Several previous studies stated that the body region most susceptible to WMSD was the neck, with pain being the most common symptom (10, 12, 15). A systematic review by AlOtaibi et al. (19) reported that the body regions with the highest prevalence of WMSD were the back (82.3%), neck (82.3%), shoulders (75.4%), and elbows/hands (30.8%). The current study shows that there were four regions with the highest proportion of musculoskeletal disorders, which were the back (68.2%) and waist (66.9%), followed by the upper neck (60.9%) and lower neck (59.6%). These areas are responsible for spinal overcapacity when working. Dental health practitioners often require a 15 to 20-degree forward bending movement, which causes muscular overload in the neck and cervical spine joints and causes contractures and pain in the neck (13). Prolonged static posture resulting from sustained muscle activity in the sternocleidomastoid or trapezius muscles may be a major etiological factor in neck pain among DPs (12, 13, 19). The repetitive stresses over a sustained period of time produce injury with load, causing creep deformation of the paraspinal muscle, possibly to the point of micro-failure (35).

In the lumbar region, musculoskeletal disorders were associated with the sitting working position in the majority of respondents (73.5%), which is in line with a study by Macrì et al. (13) which showed that dentists who mostly work in a sitting position, found the majority of pain in the lumbar and cervical regions. Incorrect sitting posture can cause a progressive reduction of the lordosis curve, which is associated with muscle weakness when maintaining an anteriorization state for a long duration. Dentists' work also often involves equipment requiring the user to maintain an unergonomic body posture, thereby stressing various anatomical locations (36).

Our study results show that the most common symptoms of musculoskeletal disorders were fatigue (53.6%) and pain (49.7%). These results align with a previous study by Phedy et al. (14) who reported that the most common symptoms of musculoskeletal disorders were fatigue (36.5%) and pain (24.9%). This study also shows that the most triggering or aggravating factors were unergonomic body postures when working (84.1%) and prolonged sitting positions (53.6%). Every joint in the body has a neutral movement zone or capacity whose movement does not require high muscle power. If the dentist makes movements outside this zone with an awkward or unergonomic posture, the symptoms of musculoskeletal disorders and the risk of injury will increase. The non-ergonomic positions in question include strained sitting positions, bending forward, tilting the shoulders, head tilts, and others. These are the etiology of WMSD in dental health practitioners (11, 13). Repeated application of low-load tasks or prolonged periods of sitting can result in a reduction of muscle failure tolerance. This can lead to stretching of the posterior spinal ligament, increasing the risk of local instability and injury to uni-segmental structures. Furthermore, this can cause an increase in shearing and bending loads on the neural arch. It is important to be aware of these potential risks and to take measures to minimize them, such as taking frequent breaks and engaging in physical activity to maintain muscle strength and endurance (35).

The current study also shows that factors that alleviate WMSD were taking a short break (71.5%) and improving body posture (53%). This is in line with previous studies where it was reported that working time, breaks between patients, and improving body posture were protective factors against WMSD among dental health practitioners (9, 11, 13, 19). Our study also shows that exercise was a protective factor against WMSD. The objective of strategies aimed at preventing injury is to ensure that the body's adaptation to stress resulting from exposure to load keeps pace with and ideally surpasses the accumulated tissue damage. To this end, while exposure to load is necessary to accumulate microtrauma, the applied loads must be removed to enable the healing and adaptation process to gradually increase the failure tolerance to the required level (35). The relationship between tissue loading and the risk of injury is optimal, and determining the optimal safety for individual tissue loading entails the application of both the art and science of medicine and biomechanics. A previous study by Letafatkar et al. (37) proved that therapeutic exercise reduces pain and disability and improves posture among dentists who experience chronic neck pain. In the current study, no association was found between the incidence of WMSD and demographic variables and job characteristics. However, previous studies reported that WMSD occurs more often in women; this is because women have low muscle tone, low strength, and hormonal factors that contribute to osteoporosis, making women more susceptible to musculoskeletal injuries (9). In addition, dental health practitioners with minimal work experience (including dental assistants) were also considered vulnerable to WMSD (9, 10, 14, 28).

A systematic review by Roll et al. examined prevention and rehabilitation techniques for musculoskeletal disorders among dental professionals. The study categorized these techniques into three main approaches: physical exercise, ergonomic training, and equipment modification (38). Evidence suggests that ergonomic training, particularly focusing on body posture and optimizing the work environment, can significantly reduce the risk of musculoskeletal disorders. Recommended strategies include maintaining a natural lumbar curve, utilizing magnification systems, adjusting dental chairs, and adopting positional and postural techniques such as avoiding static postures, alternating between standing and sitting, positioning patients at an optimal height, keeping feet parallel to the floor, repositioning feet, rotating shoulders backward, using backrests, and avoiding twisting motions. Furthermore, taking regular breaks combined with targeted stretching exercises—such as chairside directional stretching, microbreak stretches, trigger point release, and hand clasping with outward turning—can further mitigate these risks (39, 40). Additionally, studies have highlighted the benefits of using specific tools, such as magnification loupes, which improve posture and alleviate neck and back pain (41).

This study involved respondents from various dental professions, including dentists, dental nurses, and dental assistants, working in clinics and hospitals across Indonesia. Additionally, the study included dental professionals with diverse professional levels and job characteristics, aiming to provide a representative sample of dental professionals in Indonesia. However, this study has several limitations. First, being retrospective in nature, it is prone to selection bias. Second, there was an unequal distribution of respondents among dentists, dental nurses, and dental assistants. Furthermore, there was a gender imbalance, with a disproportionate female-to-male ratio, and the study did not investigate systemic factors that could contribute to musculoskeletal disorders.

Further research should focus on conducting longitudinal studies to explore the causal relationships between the identified potential risk factors—such as exercise, professional levels, and job characteristics—and the incidence of WMSDs. Additionally, it is crucial to investigate other potential contributing factors, including psychosocial conditions and systemic factors, that may play a role in the development of musculoskeletal disorders.

## Conclusion

Work-related musculoskeletal disorders (WMSDs) are highly prevalent among dental professionals (DPs), with prevalence rates reaching as high as 96%. The back and waist are the most commonly affected areas, followed by the upper and lower neck. The primary symptoms of WMSDs include muscle fatigue and pain. Key contributing factors include unergonomic body postures and prolonged sitting during work. Rest, postural correction, and exercise have been shown to be effective in preventing these disorders. WMSDs can lead to work-related disabilities, reduced productivity, compromised patient care, and increased healthcare costs. Therefore, it is crucial to educate dental health practitioners about the importance of preventive measures, including physical exercise, ergonomic training, and equipment modifications. Furthermore, future research should focus on conducting longitudinal studies to better understand the causal relationships between the identified risk factors—such as exercise, professional levels, and job characteristics—and the incidence of WMSDs.

### Limitations of the study

This study has several limitations. First, being retrospective in nature, it is prone to selection bias. Second, there was an unequal distribution of respondents among dentists, dental nurses, and dental assistants. Additionally, there was a gender imbalance, with a disproportionate female-to-male ratio, and the study did not investigate systemic factors that could contribute to musculoskeletal disorders.

## Data Availability

The raw data supporting the conclusions of this article will be made available by the authors, without undue reservation.
